# Hydrogen Sulfide Inhibits High-Salt Diet-Induced Renal Oxidative Stress and Kidney Injury in Dahl Rats

**DOI:** 10.1155/2016/2807490

**Published:** 2015-12-28

**Authors:** Pan Huang, Zhizhou Shen, Jia Liu, Yaqian Huang, Siyao Chen, Wen Yu, Suxia Wang, Yali Ren, Xiaohui Li, Chaoshu Tang, Junbao Du, Hongfang Jin

**Affiliations:** ^1^Department of Pediatrics, Peking University First Hospital, Beijing 100034, China; ^2^Lab of Electron Microscopy, Peking University First Hospital, Beijing 100034, China; ^3^Department of Cardiology, Capital Institute of Pediatrics, Beijing 100020, China; ^4^Department of Physiology and Pathophysiology, Peking University Health Science Centre, Beijing 100191, China; ^5^Key Lab of Molecular Cardiology, Ministry of Education, Beijing 100191, China

## Abstract

*Background*. The study was designed to investigate if H_2_S could inhibit high-salt diet-induced renal excessive oxidative stress and kidney injury in Dahl rats.* Methods*. Male salt-sensitive Dahl and SD rats were used. Blood pressure (BP), serum creatinine, urea, creatinine clearance rate, and 24-hour urine protein were measured. Renal ultra- and microstructures were observed. Collagen-I and -III contents the oxidants and antioxidants levels in renal tissue were detected. Keap1/Nrf2 association and Keap1 s-sulfhydration were detected.* Results*. After 8 weeks of high-salt diet, BP was significantly increased, renal function and structure were impaired, and collagen deposition was abundant in renal tissues with increased renal MPO activity, H_2_O_2_, MDA, GSSG, and ^•^OH contents, reduced renal T-AOC and GSH contents, CAT, GSH-PX and SOD activity, and SOD expressions in Dahl rats. Furthermore, endogenous H_2_S in renal tissues was decreased in Dahl rats. H_2_S donor, however, decreased BP, improved renal function and structure, and inhibited collagen excessive deposition in kidney, in association with increased antioxidative activity and reduced oxidative stress in renal tissues. H_2_S activated Nrf2 by inducing Keap1 s-sulfhydration and subsequent Keap1/Nrf2 disassociation.* Conclusions*. H_2_S protected against high-salt diet-induced renal injury associated with enhanced antioxidant capacity and inhibited renal oxidative stress.

## 1. Introduction

Numerous studies have demonstrated that high salt not only caused hypertension, but also resulted in kidney injury [1–8]. The kidney injury and fibrosis of Dahl rats induced by high salt were reported to be closely related to oxidative stress [8, 9]. However, up to now, the mechanisms responsible for high-salt-induced kidney injury have been unclear.

As the third gaseous signal molecule after nitric oxide (NO) and carbon monoxide (CO), hydrogen sulfide (H_2_S) is produced by the metabolism of sulfur-containing amino acid. The key enzymes of synthesis, cystathionine *β*-synthase (CBS), cystathionine *γ*-lyase (CSE), and mercaptopyruvate transsulphurase (MPST), are abundantly expressed in the kidney [10, 11]. H_2_S has various pathophysiological roles including relaxing blood vessels, lowering blood pressure [12, 13], anti-inflammatory response [14], antioxidative stress [15], and inhibiting proliferation of smooth muscle cells [16]. Studies showed that H_2_S might protect neurons against oxidative injury by promoting the generation of antioxidants—glutamine [17]—and participated in the regulation on hypertension induced by Ang II through antioxidative stress [18]. In hypoxia pulmonary hypertension, H_2_S could inhibit oxidative stress of lung tissue [15] and collagen deposition caused by inhibiting oxidative stress [19]. Similarly, Otunctemur et al. found that H_2_S had a protective effect on gentamicin-induced kidney injury [20]. Further study found that, in a mouse model of unilateral ureteral obstruction, H_2_S could alleviate the oxidative stress by upregulating catalase (CAT), Superoxide dismutase (SOD), and glutathione (GSH), thereby improving renal fibrosis [21]. However, whether H_2_S could protect against high-salt-induced kidney injury and what are the possible mechanisms remain unclear. Therefore, the present study was undertaken to explore the protective effects of H_2_S on high-salt-induced kidney injury in Dahl rats and its possible mechanisms.

## 2. Materials and Methods

### 2.1. Animal Grouping

Thirty 5-week-old healthy male salt-sensitive (Dahl) rats and 40 male SD rats (Charles River Laboratory Animal Technology Co., Ltd., China; License number: SCXK 2012-0001) were fed in Animal Center of Peking University First Hospital. They were randomly divided into three groups after adapting to the environment for one week. Dahl rats were divided into control group (Dahl + NS), high-salt group (Dahl + HS), and high-salt + NaHS group (Dahl + HS + NaHS), while SD rats were divided into control group (SD + NS), high-salt group (SD + HS), high-salt + NaHS group (SD + HS + NaHS), and high-salt + hydroxylamine group (SD + HS + HA), with 10 rats in each group. The rats in Dahl + NS and SD + NS groups were fed with normal diet and 0.9% normal saline was given via intraperitoneal injection daily; the rats in Dahl + HS and SD + HS groups were fed with the diet containing 8% salt and intraperitoneally injected with 0.9% normal saline every day; the rats in Dahl + HS + NaHS and SD + HS + NaHS group were fed with high-salt diet while 90 *μ*mol/kg NaHS was given by intraperitoneal injection every day [22]; and the rats in SD + HS + HA group were fed with high-salt diet while 12.5 mg/kg HA, an inhibitor of CBS, was given by intraperitoneal injection every day [23]. The NaHS and HA were prepared daily with 0.9% normal saline. All rats drank water freely throughout the experiment in 25°C constant temperature environment, maintaining 12 h/12 h circadian rhythms. This experiment strictly followed the laboratory animal welfare and the operating guide of laboratory institutions and was licensed by Peking University First Hospital Experiment and Ethics Committee.

### 2.2. Detection of Arterial Blood Pressure and Biochemical Indices in Rats

After eight weeks of experiment, 25% urethane (0.5 mL/100 g) was given to rats through intraperitoneal injection for anesthesia. Physiological multilead recorder (BL-420F, Chengdu TME, Chengdu, China) was used to record arterial blood pressure. Automatic biochemical analyzer (Hitachi 7600, Japan) was used to detect serum creatinine, creatinine clearance rate, serum urea, and 24-hour urine protein.

### 2.3. Observation of Kidney Ultramicrostructure

2 mm × 2 mm × 2 mm of renal cortical tissue was put in 3% glutaraldehyde to fix, making electron microscope specimens and observing the changes in kidney ultrastructure.

### 2.4. Observation of Renal Pathological Structure

0.2 cm of tissue specimens from kidney transverse section was taken and fixed in 4% paraformaldehyde, paraffin embedding, taking 0.5 *μ*m of section for Periodic acid-Schiff staining (Shanghai Genmed Pharmaceutical Co., Ltd., China) and Masson staining (Beijing Rocchi Biotechnology Co., Ltd., China) in strict accordance with the kit experimental steps. Leica Q550CW Image Processing and Analysis System was used for image acquisition and semiquantitative analysis of the results of kidney PAS and Masson staining. The semiquantitative analysis of kidney PAS staining was used to evaluate glomerulosclerosis. Depending on the severity of glomerulosclerosis, it is divided into four grades: (0) normal; (1) glomerulosclerosis area <25%; (2) 25%–50%; (3) 50%–75%; and (4) >75%. Ten visual fields were randomly selected for each rat to calculate the proportion of sclerotic glomeruli. Five visual fields of renal cortex were randomly selected for each rat to calculate the average index number of renal fibrosis [24].

### 2.5. Detection of Renal Collagen Content

The appropriate renal tissue was taken, adding PBS buffer (pH 7.2, 0.05 M) according to the mass volume ratio of 1 : 10 (mg/*μ*L), fully ground to renal tissue homogenate, and centrifuged at 12000 g/min for 10 min, taking the supernatant. Double antibody sandwich enzyme-linked immunosorbent assay (ELISA) was used to detect the content of collagen-I and collagen-III (American R&D Co., Ltd., USA) and hydroxyproline content was measured by colorimetry (Nanjing Jiancheng Bioengineering Institute, China). The experimental procedures were in strict accordance with the kit instructions.

### 2.6. Determination of H_2_S Content in Renal Tissue

*Preparation of Renal Tissue Homogenate*. The renal tissue of rats was taken from −70°C freezer into 1.5 mL of EP tube. 0.01 mol/L PBS solution was added according to the mass volume ratio (1/10), ground on the ice at 4°C, and centrifuged at 12000 g/min for 10 min, taking the supernatant. Free Radical Detection Analyzer TBR4100 (World Precision Instruments, Shanghai, China) was used to measure H_2_S content in the supernatant of renal tissue homogenates [25]. Firstly, 2 mm of ISO-H_2_S-100 sensor (ISO-H_2_S-2, WPI, Shanghai, China) was placed in PBS buffer solution (pH 7.2, 0.05 M) for polarization. When the stable reference current appeared (usually as 100–2000 PA), the sensor was calibrated. Approximate 10–15 mm of the top of the sensor was immersed in 20 mL of PBS buffer solution (pH 7.2, 0.05 M), until the stable current appeared on the display. Then, the detecting sensors were successively inserted in six kinds of different concentrations of Na_2_S solution formulated by PBS buffer (5 *μ*L, 10 *μ*L, 20 *μ*L, 40 *μ*L, 80 *μ*L, and 160 *μ*L). The current output would rise rapidly to a plateau after each sample was added. As long as the current reached a plateau, the next sample was detected. Then, the calibration curve was made based on the output signal (pA) and the corresponding H_2_S concentration (*μ*mol/L). There was about 10–15 mm of the sensor that was immersed in each sample. H_2_S content of each sample was calculated according to pA- H_2_S concentration calibration curve.

### 2.7. Detection of Oxidative Stress Indices of Renal Tissue

The right amount of renal tissue was taken; PBS buffer (pH 7.2, 0.05 M) was added according to the mass volume ratio of 1 : 10 (mg/*μ*L), fully ground to renal tissue homogenate, and centrifuged at 12000 g/min for 10 min, taking the supernatant. Biochemical colorimetry was used to detect the following indices: SOD, myeloperoxidase (MPO), CAT, glutathione peroxidase (GSH-PX) activity and oxidized glutathione (GSSG), GSH, hydrogen peroxide (H_2_O_2_), malondialdehyde (MDA), hydroxyl radical (OH) content, and total antioxidant capacity (T-AOC). The experimental procedures were in strict accordance with the kit instructions (Nanjing Jiancheng Bioengineering Institute, China).

### 2.8. Protein Levels of SOD2 and SOD1 of Renal Tissue Detected by Western Blot

The renal tissues were homogenized at 4°C by adding 1x tissue lysates according to the mass volume ratio of 1 : 10 (mg/*μ*L) and then centrifuged at 12000 g/min for 10 min, taking the supernatant. The equal amounts of protein mixture samples were added into each channel for polyacrylamide gel electrophoresis and then transferred onto a membrane. After closing by milk at room temperature for 1 h, primary antibodies SOD1 (Stressgen, USA), SOD2 (Stressgen, USA), and *β*-actin (Santa Cruz Biotechnology) were added, incubated at 4°C overnight, and washed by TTBS, 10 min/times, for a total of four times. Then, HRP-labeled secondary antibody (Sigma, USA) was added, incubated at room temperature for 1 h, washed by TTBS, 10 min/times, for a total of four times, and then incubated with chemiluminescent reagents for 1 min before being exposed, developed, and fixed. AlphaImager gel imaging system was used to scan protein bands and measure optical density of protein bands, and *β*-actin was used as interior reference to correct [26].

### 2.9. The Association of Keap1 and Nrf2 Detected with Coimmunoprecipitation

The coimmunoprecipitation was performed as described previously [27]. Briefly, the renal tissues were fully ground on ice with RIPA lysis buffer (20 mm Tris, 150 mm NaCl, 1% Triton X-100, EDTA, and proteinase and phosphatase inhibitors) and then centrifuged at 12000 g/min for 20 min at 4°C. The equal amount of protein supernatant after protein quantization was incubated overnight at 4°C with Keap1 or Nrf2 antibody. Protein A/G-magnetic beads were added at 4°C and incubated for 4 h and then at 4°C, centrifuged at 10000 g/min for 10 min. Protein A/G-magnetic beads were washed with PBS buffer (pH 7.2, 0.05 M) for 3 times and then boiled for 10 min at 100°C mixed with 2x loading buffer. The proteins were separated in 10% SDS-PAGE and detected with Nrf2 (Enzolife, USA) or Keap1 antibody (Cell Signaling Technology, USA) by Western blotting.

### 2.10. s-Sulfhydration Assay of Keap1

Keap1 s-sulfhydration was performed by the modified biotin switch assay as described previously [28]. The renal tissues were homogenized at 4°C with tissue lysis buffer and centrifuged at 12000 g/min for 10 min. The tissue lysates were incubated with blocking buffer (lysis buffer supplemented with 2.5% SDS and 20 mM S-methyl methanethiosulfonate) at 50°C for 20 min with frequent vortexing; then acetone was added for removing S-methyl methanethiosulfonate and proteins were precipitated at −20°C for 2 h. After acetone removal, the proteins were resuspended in tissue lysis buffer and incubated with 25 *μ*L of EZ-Link iodoacetyl-PEG2 biotin (10 mg/mL) for 12 h at 4°C. Biotinylated proteins were precipitated by 30 *μ*L of ultralink immobilized NeutrAvidin for 4 h at 4°C. After supernatant removal, the protein beads were washed with PBS buffer (pH 7.2, 0.05 M) for 3 times and boiled for 10 min at 100°C mixed with loading buffer without *β*-mercaptoethanol. Then, the biotinylated proteins were detected with Keap1 antibody by Western blotting [28].

### 2.11. Statistical Analysis

SPSS13.0 software was used for statistical analysis. The results are expressed as mean ± standard error, the means between the two groups were compared by independent sample *t*-test, and the means among many groups were compared by one-way ANOVA. *P* < 0.05 was considered significant.

## 3. Results

### 3.1. High-Salt Diet Induced the Significant Increase of Blood Pressure and Renal Impairment in Dahl Rats

**Figure 1:**
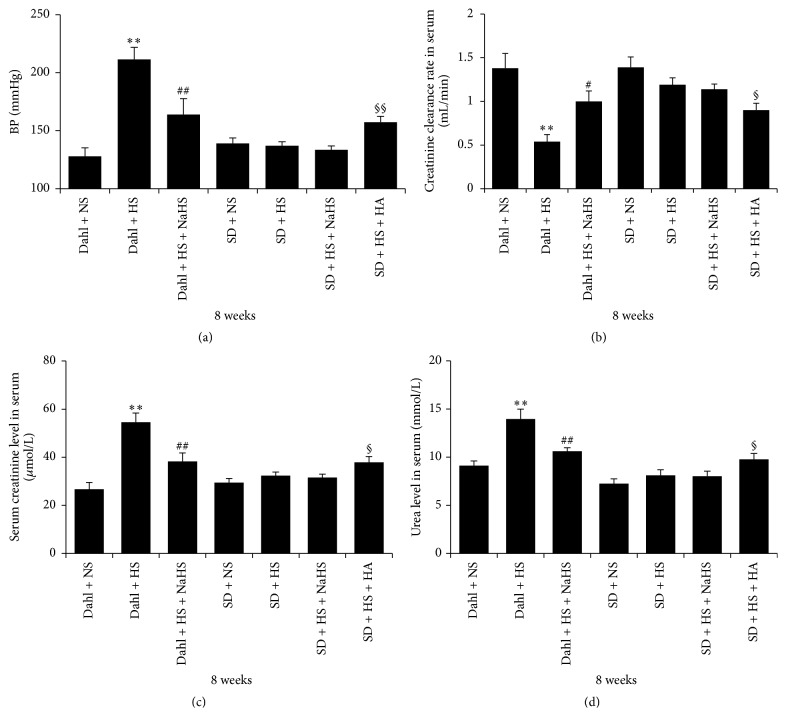
H_2_S improved blood pressure and renal function of Dahl rats induced by high salt. (a) BP and (b and d) renal function indices (mean ± SE, *n* = 10). ^*∗∗*^ *P* < 0.01 versus Dahl + NS; ^#^ *P* < 0.05, ^##^ *P* < 0.01 versus Dahl + HS; ^§^ *P* < 0.05 versus SD + HS; and ^§§^ *P* < 0.01 versus SD + HS.

Compared with the rats of Dahl + NS, blood pressure of rats in Dahl + HS group was significantly increased (*P* < 0.01, Figure 1(a)); renal function was decreased, as reflected by the reduced creatinine clearance rate (*P* < 0.01, Figure 1(b)) and the increased content of serum creatinine and serum urea (*P* < 0.01, Figures 1(c) and 1(d)); however, compared with the rats of SD + NS group, blood pressure and renal function of SD rats did not change in SD + HS group. There was no significant change in creatinine clearance rate, serum creatinine, and serum urea between rats of SD + NS group and SD + HS group (*P* > 0.05, Figures 1(a)–1(d)).

### 3.2. High-Salt Diet Caused the Increase in Urine Protein and Renal Structural Damage in Dahl Rats

**Figure 2:**
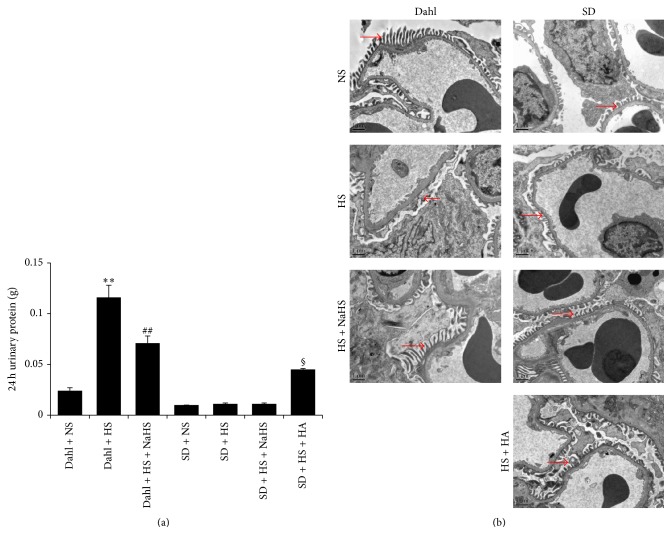
H_2_S improved proteinuria and renal structural damage of Dahl rats induced by high salt. (a) 24 h urinary protein (mean ± SE, *n* = 10). ^*∗∗*^ *P* < 0.01 versus Dahl + NS; ^##^ *P* < 0.01 versus Dahl + HS; and ^§^ *P* < 0.05 versus SD + HS. (b) Electron microscope of kidney changes in kidney ultrastructure (TEM 8000x). →: foot process of glomerular podocytes.

**Figure 3:**
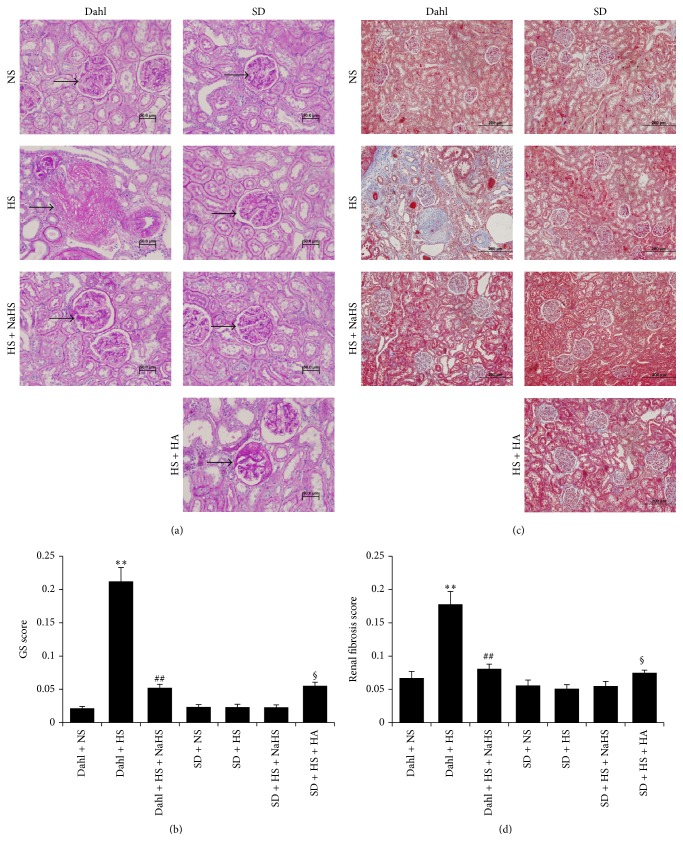
PAS and Masson staining showed the pathological structural changes in rat kidney in each group. (a) PAS staining of kidney (400x). (b) Semiquantitative measurement of glomerular sclerosis by PAS staining (mean ± SE, *n* = 10). (c) Kidney Masson staining (200x). (d) Semiquantitative measurement of renal fibrosis by Masson staining. ^*∗∗*^ *P* < 0.01 versus Dahl + NS; ^##^ *P* < 0.01 versus Dahl + HS; and ^§^ *P* < 0.05 versus SD + HS. →: glomerulus.

Compared with the rats of Dahl + NS group, 24 h urine protein of rats in Dahl + HS group was significantly increased (*P* < 0.01, Figure 2(a)), and the electron microscopic result of renal tissue showed that the foot process of glomerular podocytes had an extensive fusion or even disappeared (Figure 2(b)). PAS staining of renal tissue showed that there was an obvious glomerular sclerosis in rats of Dahl + HS group (*P* < 0.01, Figures 3(a) and 3(b)). Masson staining of renal tissue showed that there was renal fibrosis in rats of Dahl + HS group (*P* < 0.01, Figures 3(c) and 3(d)).

### 3.3. High-Salt Diet Induced Decrease in H_2_S Content of Renal Tissue in Dahl Rats

**Figure 4:**
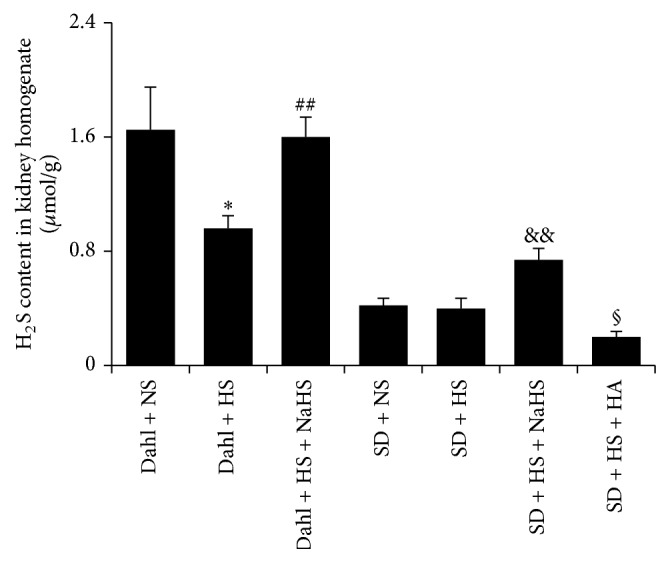
Changes in H_2_S content of rat renal tissue in each group (mean ± SE, *n* = 10). ^*∗*^ *P* < 0.01 versus Dahl + NS; ^##^ *P* < 0.01 versus Dahl + HS; ^&&^ *P* < 0.01 versus SD + HS; and ^§^ *P* < 0.05 versus SD  +  HS.

Compared with rats in Dahl + NS group, H_2_S content of renal tissue of rats in Dahl + HS group was significantly decreased (*P* < 0.05, Figure 4).

### 3.4. H_2_S Improved Blood Pressure and Renal Function of Dahl Rats Induced by High Salt

Compared with Dahl + HS rats, blood pressure of rats in Dahl + HS + NaHS group was significantly decreased (*P* < 0.01, Figure 1(a)); renal function was significantly improved as demonstrated by the obviously increased creatinine clearance rate and the significantly decreased content of serum creatinine and serum urea (*P* < 0.05, *P* < 0.01, Figures 1(b)–1(d)) after the treatment with NaHS. However, blood pressure and renal function did not differ between rats of SD + HS group and SD + HS + NaHS group (*P* > 0.05, Figures 1(a)–1(d)). While, compared with SD + HS rats, blood pressure of SD + HS + HA rats was significantly increased (*P* < 0.01, Figure 1(a)), renal function was significantly damaged with the obviously decreased creatinine clearance rate and the significantly increased content of serum creatinine and serum urea (*P* < 0.05, Figures 1(b)–1(d)) after the treatment with HA.

### 3.5. H_2_S Improved 24 h Urine Protein and Renal Structural Damage of Dahl Rats Induced by High Salt

Compared with Dahl + HS rats, 24 h urine protein of Dahl rats in high-salt group was decreased after the treatment with NaHS (*P* < 0.01, Figure 2(a)), and the electron microscope result of renal tissue showed that the foot process fusion of glomerular podocytes had a significant relief (Figure 2(b)). PAS staining and semiquantitative analysis showed that there was a significant relief in glomerular sclerosis (*P* < 0.01, Figures 3(a) and 3(b)). Masson staining and semiquantitative analysis showed that there was a significant relief in renal fibrosis (*P* < 0.01, Figures 3(c) and 3(d)). However, in SD rats, NaHS had no significant effect on 24 h urine protein and renal structure, but, compared with SD + HS rats, HA significantly increased 24 h urine protein (*P* < 0.05, Figure 2(a)) and damaged renal structure (Figure 2(b)).

### 3.6. H_2_S Reduced the Renal Collagen Content in Dahl Rats Induced by High Salt

**Figure 5:**
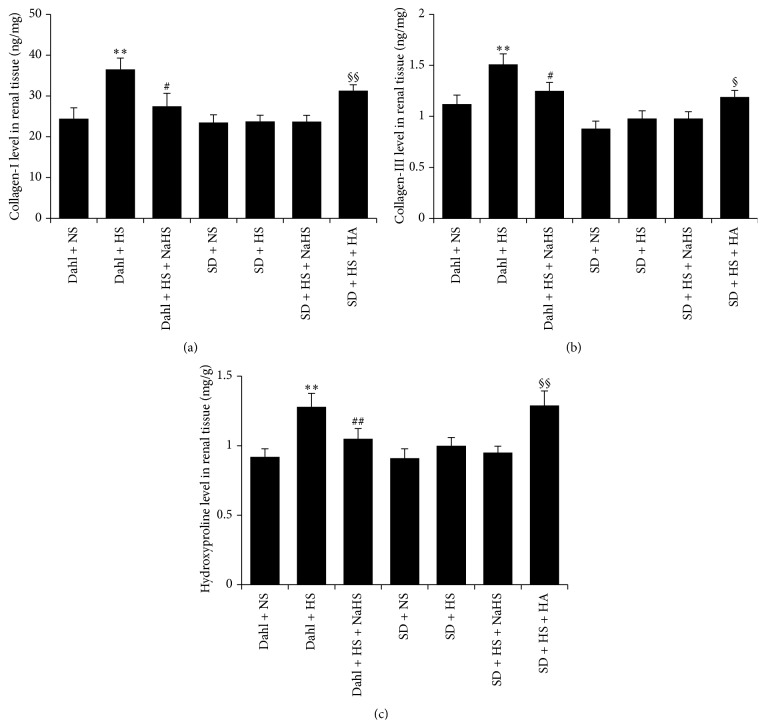
H_2_S reduced the content of collagen of renal tissue in Dahl rats induced by high salt. (a) Collagen-I content of rat renal tissue in each group. (b) Collagen-III content of rat renal tissue in each group. (c) Hydroxyproline content of rat renal tissue in each group (mean ± SE, *n* = 10). ^*∗∗*^ *P* < 0.01 versus Dahl + NS; ^#^ *P* < 0.05, ^##^ *P* < 0.01 versus Dahl + HS; ^§^ *P* < 0.05 versus SD + HS; and ^§§^ *P* < 0.01 versus SD + HS.

Compared with Dahl + NS group, the contents of collagen-I, collagen-III, and hydroxyproline of renal tissue in Dahl + HS group were significantly increased (*P* < 0.01, Figures 5(a)–5(c)). Compared with Dahl + HS group, however, the contents of collagen-I, collagen-III, and hydroxyproline of renal tissue in Dahl + HS + NaHS group were significantly reduced (*P* < 0.05, *P* < 0.01, Figures 7(a)–7(c)). Compared with rats of SD + HS group, NaHS had no significant effect on the contents of collagen-I, collagen-III, and hydroxyproline in rat renal tissue of SD + HS + NaHS group (*P* > 0.05, Figures 5(a)–5(c)), but HA significantly increased the contents of collagen-I, collagen-III, and hydroxyproline in rat renal tissue of SD + HS + NaHS group (*P* < 0.05, *P* < 0.01, Figures 5(a)–5(c)).

### 3.7. H_2_S Reduced Renal MDA, H_2_O_2_, ^•^OH, and GSSG Content and MPO Activity in Dahl Rats with High-Salt Diet

**Figure 6:**
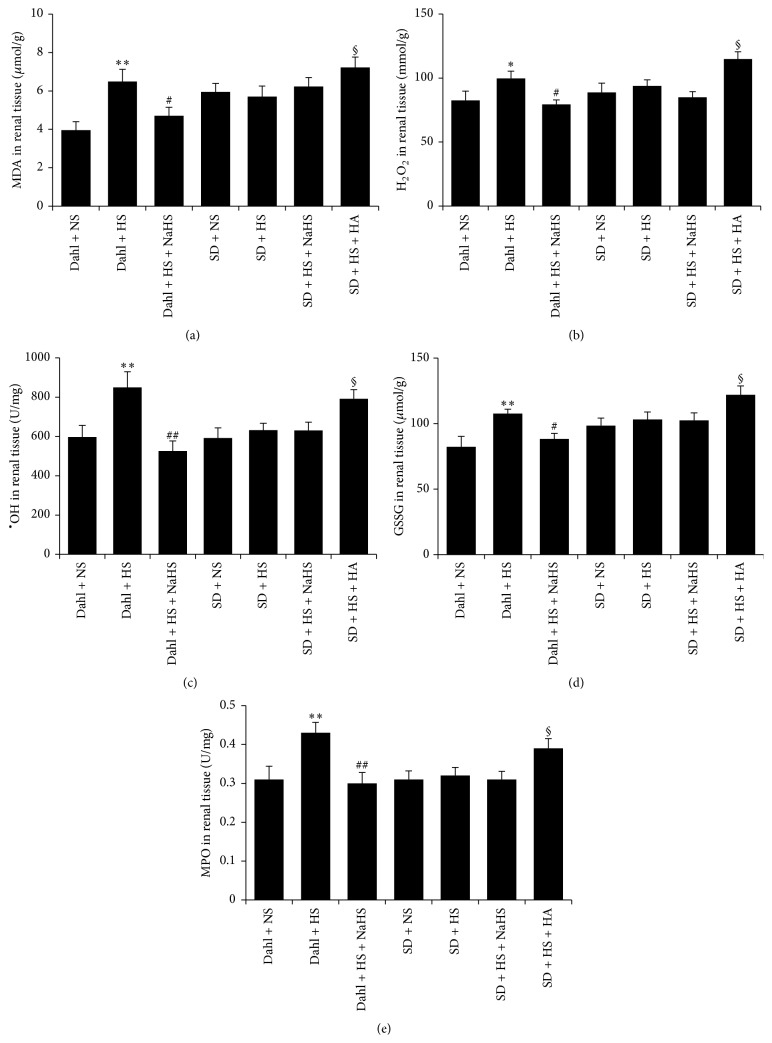
H_2_S inhibited oxidative stress of Dahl rat renal tissue induced by high salt. (a) MDA content of rat renal tissue in each group. (b) H_2_O_2 _content of rat renal tissue in each group. (c) ^•^OH content of rat renal tissue in each group. (d) GSSG content of rat renal tissue in each group. (e) MPO activity of rat renal tissue in each group (mean ± SE, *n* = 10). ^*∗*^ *P* < 0.05, ^*∗∗*^ *P* < 0.01 versus Dahl + NS; ^#^ *P* < 0.05, ^##^ *P* < 0.01 versus Dahl + HS; and ^§^ *P* < 0.05 versus SD + HS.

Compared with Dahl + NS group, the contents of MDA, H_2_O_2_, ^•^OH, and GSSG of renal tissues in Dahl + HS group were significantly increased (*P* < 0.01, *P* < 0.05, Figures 6(a)–6(d)), and MPO activity was increased (*P* < 0.01, Figure 6(e)). However, compared with Dahl + HS group, the contents of MDA, H_2_O_2_, ^•^OH, and GSSG of renal tissue in Dahl + HS + NaHS group were significantly decreased (*P* < 0.05, *P* < 0.01, Figures 6(a)–6(d)), and MPO activity was decreased (*P* < 0.01, Figure 6(e)). Compared with SD + NS group, however, the contents of MDA, H_2_O_2_, ^•^OH, and GSSG of renal tissue in SD + HS group did not alter (*P* > 0.05, Figures 6(a)–6(d)), nor the MPO activity (*P* > 0.05, Figure 6(e)). Compared with SD + HS group, the contents of MDA, H_2_O_2_, ^•^OH, and GSSG of renal tissue did not change in SD + HS + NaHS group (*P* > 0.05, Figures 6(a)–6(d)), and there was no increase in MPO activity (*P* > 0.05, Figure 6(e)), while the contents of MDA, H_2_O_2_, ^•^OH, and GSSG of renal tissue were increased in SD + HS + HA group (*P* < 0.05, Figures 6(a)–6(d)), and there was a significant increase in renal MPO activity (*P* < 0.05, Figure 6(e)).

### 3.8. H_2_S Enhanced T-AOC and Increased GSH Content and the Activity of CAT, GSH-PX, and SOD of Dahl Rat Renal Tissue Induced by High Salt

**Figure 7:**
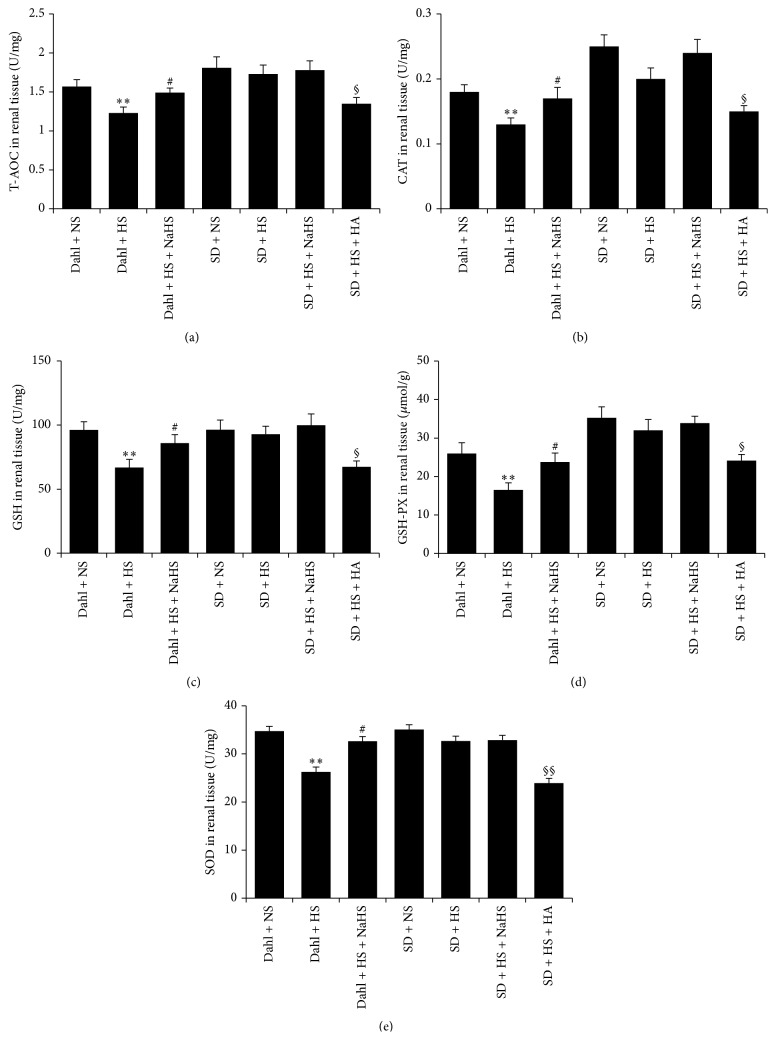
H_2_S enhanced the antioxidant capacity of Dahl rat renal tissue induced by high salt. (a) T-AOC of rat renal tissue in each group. (b) CAT activity of rat renal tissue in each group. (c) GSH activity of rat renal tissue in each group. (d) GSH-Px activity of rat renal tissue in each group. (e) SOD activity of rat renal tissue in each group (mean ± SE, *n* = 10). ^*∗∗*^ *P* < 0.01 versus Dahl + NS; ^#^ *P* < 0.05 versus Dahl + HS; ^§^ *P* < 0.05 versus SD + HS; and ^§§^ *P* < 0.01 versus SD + HS.

Compared with Dahl + NS group, T-AOC of renal tissue in rats of Dahl + HS group was decreased (*P* < 0.01, Figure 7(a)), and antioxidant enzymes CAT, GSH-PX, and SOD activities and GSH content were significantly reduced (*P* < 0.05, *P* < 0.01, Figures 7(b)–7(e)). But compared with Dahl + HS group, T-AOC of renal tissue in Dahl + HS + NaHS group was potently enhanced (*P* < 0.05, Figure 7(a)) and renal CAT, GSH-PX, and SOD activities and GSH content were significantly increased (*P* < 0.05, Figures 7(b)–7(e)). However, compared with SD + NS group, there was no significant change in T-AOC, GSH content and the activity of CAT, GSH-PX, and SOD of renal tissue in SD + HS group (*P* > 0.05, Figure 7), and renal tissue T-AOC, GSH content and the activity of CAT, GSH-PX, and SOD did not differ between SD + HS group and SD + HS + NaHS group (*P* > 0.05, Figure 7), but renal tissue T-AOC, GSH content and the activity of CAT, GSH-PX, and SOD in SD + HS + HA group were significantly decreased as compared with SD + HS group (*P* < 0.05, *P* < 0.01, Figure 7).

### 3.9. H_2_S Upregulated the Protein Expression of SOD1 and SOD2 of Dahl Rat Renal Tissue Induced by High Salt

**Figure 8:**
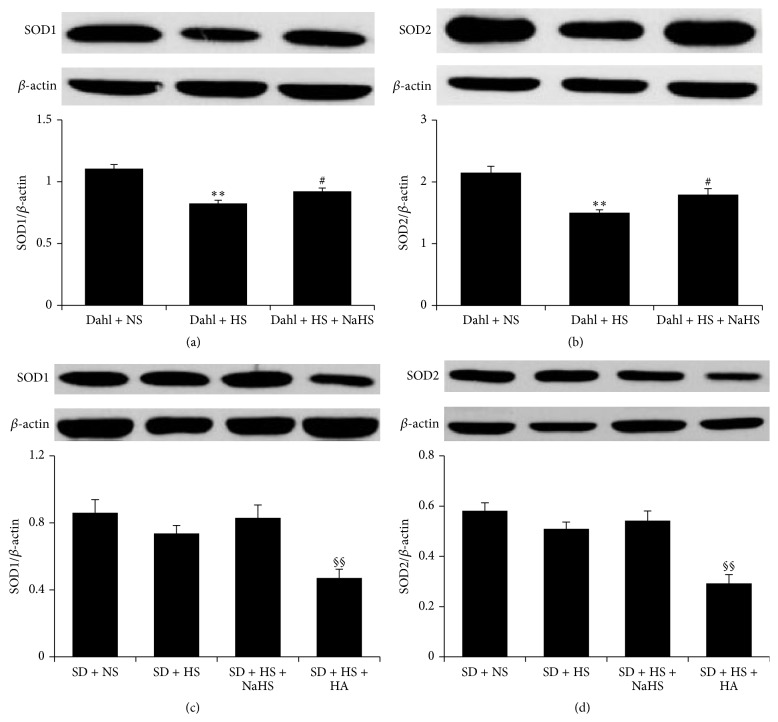
Protein expression of SOD1 and SOD2 in renal tissues of Dahl rats and SD rats. (a) SOD1 and (b) SOD2 protein expressions in renal tissues of Dahl rats. (c) SOD1 and (d) SOD2 protein expressions in renal tissues of SD rats (mean ± SE, *n* = 10). ^*∗∗*^ *P* < 0.01 versus Dahl + NS; ^#^ *P* < 0.05 versus Dahl + HS; ^§^ *P* < 0.05 versus SD + HS; and ^§§^ *P* < 0.01 versus SD + HS.

Compared with Dahl + NS group, the expression of SOD1 and SOD2 protein in renal tissue of Dahl + HS group was reduced (*P* < 0.01, Figures 8(a) and 8(b)). However, compared with Dahl + HS group, the expression of SOD1 and SOD2 protein in renal tissue of Dahl + HS + NaHS group was enhanced (*P* < 0.05, Figures 8(a) and 8(b)). Among the rats of SD + NS, SD + HS, and SD + HS + NaHS groups, there was no significant change in the expression of SOD protein of renal tissue (*P* > 0.05, Figures 8(c) and 8(d)). However, compared with SD + HS group, the expression of SOD1 and SOD2 protein of renal tissue in SD + HS + HA group was decreased (*P* < 0.01, Figures 8(c) and 8(d)).

### 3.10. H_2_S Reduced Association of Keap1 with Nrf2 and Enhanced Keap1 s-Sulfhydration in Renal Tissue of Dahl Rats with High-Salt Diet

**Figure 9:**
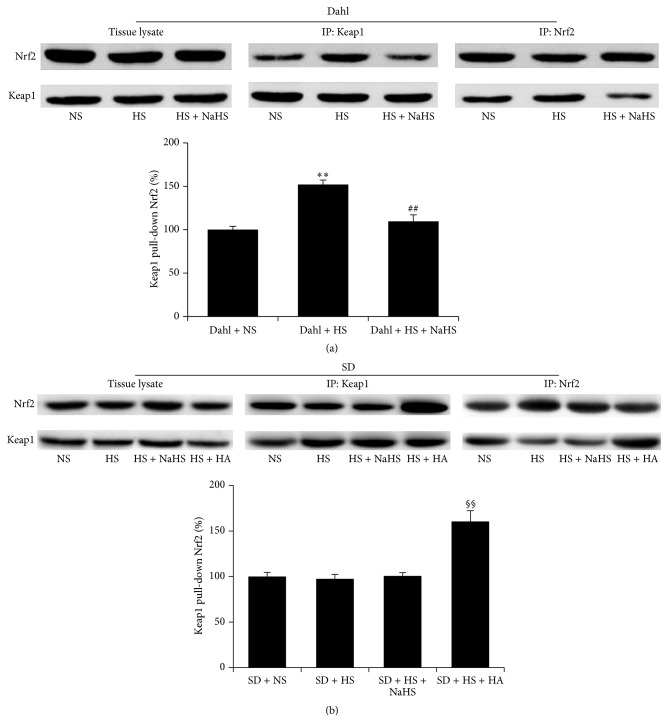
The effect of H_2_S on the association of Keap1 with Nrf2 in renal tissues of Dahl rats and SD rats. (a) The association of Keap1 with Nrf2 in renal tissues of Dahl rats. (b) The association of Keap1 with Nrf2 in renal tissues of SD rats (mean ± SE, *n* = 10). ^*∗∗*^ *P* < 0.01 versus Dahl + NS; ^##^ *P* < 0.05 versus Dahl + HS; and ^§§^ *P* < 0.01 versus SD + HS.

**Figure 10:**
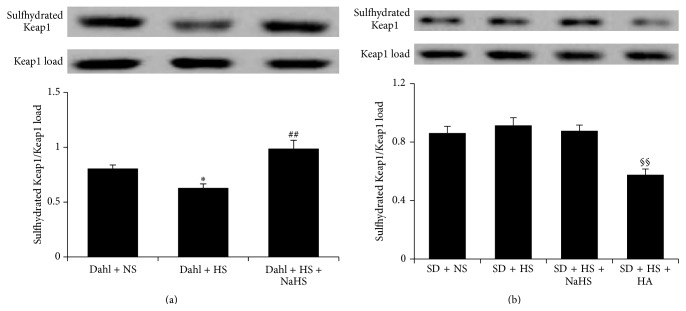
The effect of H_2_S on Keap1 s-sulfhydration in renal tissues of Dahl rats and SD rats. (a) Keap1 s-sulfhydration in renal tissues of Dahl rats. (b) Keap1 s-sulfhydration in renal tissues of SD rats (mean ± SE, *n* = 10). ^*∗*^ *P* < 0.05 versus Dahl + NS; ^##^ *P* < 0.01 versus Dahl + HS; and ^§§^ *P* < 0.01 versus SD + HS.

Compared with Dahl + NS group, the association of Keap1 with Nrf2 in the renal tissue of rats was increased in Dahl + HS group (*P* < 0.01, Figure 9(a)), but Keap1 s-sulfhydration in the renal tissue of rats in Dahl + HS group was reduced (*P* < 0.05, Figure 10(a)). However, NaHS treatment significantly reduced the association of Keap1 with Nrf2 (*P* < 0.01, Figure 9(a)) and enhanced Keap1 s-sulfhydration in the renal tissues of rats (*P* < 0.01, Figure 10(a)) in Dahl + HS group. There were no significant differences in the associated Keap1 with Nrf2 and Keap1 s-sulfhydration in the renal tissues of rats among SD + NS, SD + HS, and SD + HS + NaHS groups (*P* > 0.05, Figures 9(b) and 10(b)). However, compared with SD + HS group, the association of Keap1 with Nrf2 in the renal tissues of rats in SD + HS + HA group was increased (*P* < 0.01, Figure 9(b)) and Keap1 s-sulfhydration in renal tissue was reduced (*P* < 0.01, Figure 10(b)).

## 4. Discussion

In recent years, studies on the effect of excessive intake of salt in diet on human health have received considerable attentions. Previous studies showed that the high-salt diet was closely associated with hypertension and could cause kidney damage [29]. As an important organ, the kidney is involved in the regulation of water-electrolyte metabolism and blood pressure. Hence, its functional and structural damage will exacerbate the process of hypertension and cause serious complications. A large number of renal transplantation experiments of animal and human have confirmed that kidney is a key factor for hypertension caused by high salt [30–36]. Therefore, the study on kidney injury caused by high salt is crucial to preventing the occurrence of hypertension due to high salt.

In the previous experiment on Dahl rats induced by high salt, it was reported that antioxidants vitamin E and vitamin C could effectively reverse the kidney damage caused by high-salt diet. Studies have shown that, as a strong reducing agent, H_2_S has antioxidative stress effect [19, 20, 37]. Therefore, this experiment was undertaken to study the protective mechanisms by which H_2_S regulated kidney damage of Dahl rats caused by high salt.

In the present study, we used high-salt diet to stimulate Dahl and SD rats for eight weeks, and blood pressure of Dahl rat in high-salt group was significantly increased with proteinuria. There was an obvious renal structural damage. Interestingly, the study showed that H_2_S content of Dahl rat renal tissue in high-salt group was decreased while there was no change in the above indices between SD rats with and without high-salt diet. The results suggested that the decreased H_2_S content of renal tissue might be involved in the mechanism for high-salt-induced hypertension and kidney damage in Dahl rats. To confirm this point, the high-salt diet was given to Dahl and SD rats with NaHS treatment daily by intraperitoneal injection and to SD rats with HA treatment daily by intraperitoneal injection. The results showed that blood pressure in rats of Dahl + HS + NaHS group was decreased significantly; proteinuria and renal structural damage were significantly reduced, but these indices of SD rats did not change no matter NaHS was used or not while the blood pressure and proteinuria of SD + HS + HA group were increased significantly with renal structural damage. Previous studies showed that H_2_S/CBS pathway of renal tissue was downregulated in the formation of hypertension [11]. Studies also showed that H_2_S could effectively improve renal function and kidney damage in kidney transplant experiments [38–40]. Our study firstly confirmed that H_2_S played a protective role in high-salt-induced kidney injury in Dahl rats.

Up to now, the mechanism by which H_2_S protected against the high-salt-induced kidney function and structure has not been clear. Oxidative stress refers to a process of the imbalance between oxidation and antioxidation which can lead to the excessive accumulation of reactive oxygen species and reactive nitrogen* in vivo* or in the cell, causing the oxidative damage. ROS includes superoxide anion (O_2_^−^), ^•^OH^−^, and H_2_O_2_, and RNS includes NO, nitrogen dioxide, and peroxynitrite. There are two types of enzymatic and nonenzymatic antioxidant systems in the body, and the former includes SOD, CAT, and GSH-PX, while the latter includes vitamin C, vitamin E, and glutathione peptide [41, 42]. Studies have shown that H_2_S has antioxidative stress effect [21, 43, 44]. We further detected kidney oxidative stress indices in high-salt-induced kidney animal model and found that high salt caused oxidative stress of Dahl rat renal tissue, as demonstrated by the decreased T-AOC, GSH and the decreased activity of CAT, GSH-PX, and SOD, while the levels of MDA, H_2_O_2_, ^•^OH, and GSSG were increased, and MPO activity was also increased. The results suggested that high salt could lead to oxidative stress of Dahl rat renal tissue. After giving NaHS, an H_2_S donor, into Dahl rats, the renal T-AOC and GSH content were enhanced, the renal activity of CAT, GSH-PX, and SOD increased, and expression of SOD1 and SOD2 protein was upregulated, while the content of MDA, H_2_O_2_, ^•^OH, GSSG, and MPO activity decreased. The results suggested that H_2_S could increase the renal antioxidative ability and reduce the oxidative injury.

As well known, Keap1/Nrf2 is one of the main pathways for antioxidative system. It regulates expression of many antioxidant enzymes and plays a pivot role in the homeostasis of oxidative and antioxidative responses [27, 45–47]. Keap1 is an inhibitor of the transcription factor Nrf2 and located in the cytoplasm, binding with Nrf2 to prevent its translocation to the nucleus Keap1/Nrf2 dissociation, which results in Nrf2 nuclear translocation and increases the expression of target antioxidant enzymes such as SOD, further playing the role of antioxidation. In the present study, we found that the increase in association of Keap1 with Nrf2 in the Dahl rats with high-salt diet was reversed by treatment with H_2_S donor. Furthermore, the increase in combination of Keap1 with Nrf2 in the renal tissues of Dahl + HS rats was mimicked by giving HA, an inhibitor of endogenous H_2_S generation, in the renal tissues of SD rats with high-salt diet. The above results suggested that H_2_S might decrease the combination of Keap1 with Nrf2, thereby releasing the free Nrf2 and subsequently activating Nrf2. Regarding the mechanism by which H_2_S reduced the association of Keap1 with Nrf2, previous studies have reported that Keap1 s-sulfhydration could destroy the combination of Keap1 with Nrf2 and then enhance the Nrf2 nuclear translocation [27, 46]. While our results showed that chronic supplement H_2_S donor enhanced Keap1 s-sulfhydration in the renal tissues of Dahl + HS rats, CBS inhibitor HA increased Keap1 s-sulfhydration in the renal tissues of SD + HS rats. However, further studies are needed to deepen the understanding of the mechanism by which H_2_S plays an important role in the antioxidation.

## 5. Conclusions

Renal oxidative injury and collagen deposition were observed in Dahl rats with hypertension induced by high salt. H_2_S upregulated the expression of SOD protein, improved antioxidant capacity, removed ROS, and thereby improved renal function and renal structural injury. s-Sulfhydration of Keap1 and thereby dissociation of Keap1 with Nrf2 might be involved, the mechanisms by which H_2_S enhanced the expression of antioxidant enzyme. In conclusion, H_2_S could reduce the high-salt-induced renal injury in association with inhibiting renal oxidative stress in Dahl rats. Keap1/Nrf2 pathway might mediate the antioxidant role of H_2_S.
